# Association of Surgical Margin Status with Oncologic Outcome in Patients Treated with Breast-Conserving Surgery

**DOI:** 10.3390/curroncol29120726

**Published:** 2022-11-27

**Authors:** Sumin Chae, Sun Young Min

**Affiliations:** 1 Department of Medicine, Graduate School, Kyung Hee University, Seoul 02447, Republic of Korea; 2 Department of Surgery, Kyung Hee University Medical Center, Kyung Hee University College of Medicine, Seoul 02447, Republic of Korea

**Keywords:** breast-conserving surgery, close resection margin, residual disease, locoregional recurrence

## Abstract

We aimed to compare the prognosis of patients with close resection margins after breast-conserving surgery (BCS) with that of patients with negative margins and identified predictors of residual disease. A total of 542 patients with breast cancer who underwent BCS between 2003 and 2019 were selected and divided into the close margin (114 patients) and negative margin (428 patients) groups. The median follow-up period was 72 (interquartile range, 42–113) months. Most patients received radiation therapy (RTx) and systemic therapy according to their stage and molecular subtype. The 10-year locoregional recurrence-free survival rates of the close and negative margin groups were 88.2% and 95.5%, respectively (*p* = 0.001). Multivariable analysis showed that adjuvant RTx and margin status after definitive surgery were significantly associated with locoregional recurrence. Of the 57 patients who underwent re-excision, 34 (59.6%) had residual disease. Multivariable analysis revealed that a histological type of positive or close margins and multifocality were independent predictive factors for residual disease. Although the current guidelines suggest that *no ink on tumor* is an adequate margin after BCS, a close resection margin may be associated with locoregional failure. The treatment strategy for close resection margins after BCS should be based on individual clinicopathological features.

## 1. Introduction

The global 5-year incidence of breast cancer exceeds 43 million cases. According to GLOBOCAN 2018, breast cancer is one of the most common cancers in women and is the leading cause of female cancer mortality [1,2]. The use of total mastectomy with breast reconstruction or robot-assisted mastectomy for breast cancer treatment has increased over time to improve patient quality of life by minimizing psychosocial stress and postoperative complications [1,2,3]. However, breast-conserving surgery (BCS), also known as lumpectomy, remains an important modality of early breast cancer treatment. According to several randomized controlled trials, there is no difference in disease-free survival and overall survival between patients undergoing BCS followed by adjuvant radiation therapy (RTx) and those undergoing mastectomy [4,5,6]. However, positive or close resection margins after BCS increase the risk of local recurrence (LR) [7,8]. LR is associated with an increased risk of distant recurrence and worse survival [9]. Consequently, the goals of BCS are to achieve a clear margin and to minimize the risk of recurrence. The Society of Surgical Oncology and American Society for Radiation Oncology has published consensus guidelines on margins for BCS followed by RTx in early stage breast cancer [10,11]. They concluded that *no ink on tumor* should be the standard for an adequate margin status in stages I and II invasive breast cancer. Although re-excision is generally recommended for positive margins, several studies have reported an absence of residual cancer in 37–70% of patients with positive margins on histological examination of re-excision specimens [12,13,14,15,16]. In contrast, studies have reported rates of residual disease with negative but close margins of up to 55% [17,18]. Therefore, adequate margins after BCS remain controversial, and a close margin often makes the decision to perform re-excision challenging. The purpose of this study was to evaluate the association of final margin status with locoregional recurrence (LRR) in patients who underwent BCS and to identify factors associated with residual disease after re-excision for positive or close margins.

## 2. Materials and Methods

**Figure 1:**
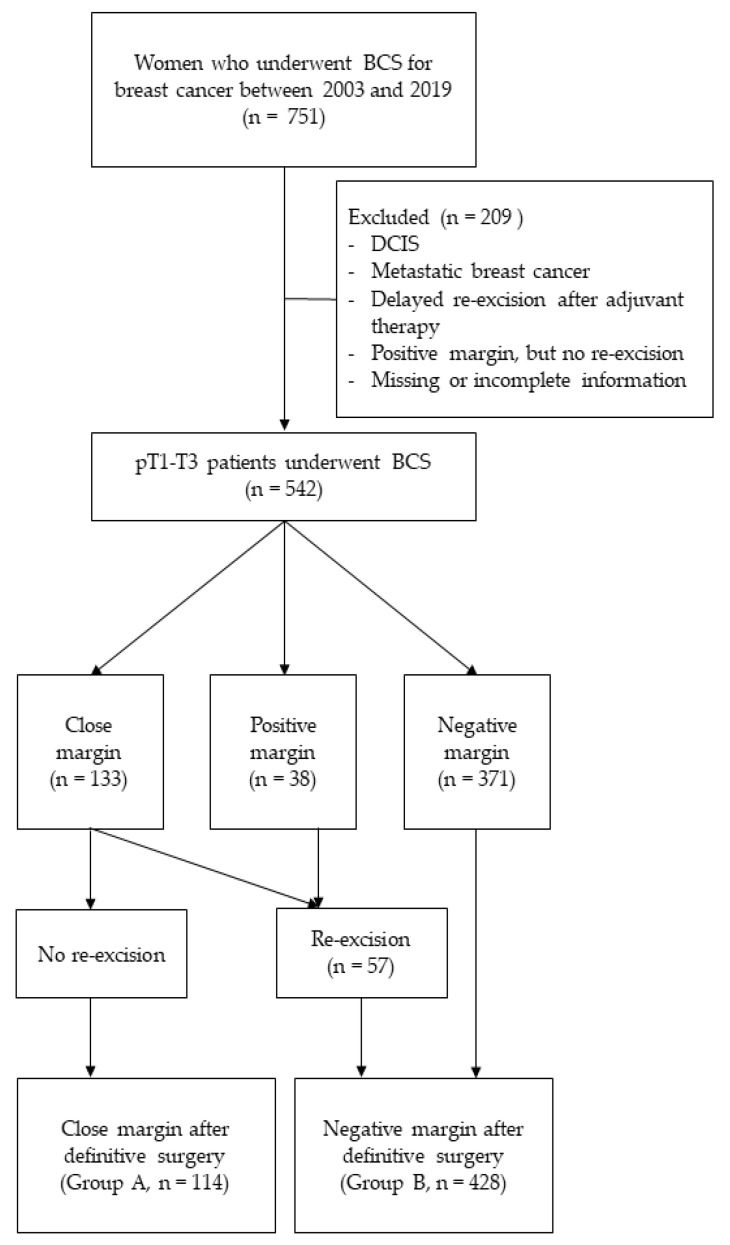
Patient selection flowchart.

This was a retrospective cohort study. Patients with newly diagnosed primary breast cancer who underwent BCS between 2003 and 2019 at the Kyung Hee University Medical Center (KHUMC) were considered. The patients’ medical charts were reviewed, and 751 patients with breast cancer who were treated with BCS were identified. The following patients were excluded from the analysis: those with pathologically staged Tis or metastatic cancer, those who did not undergo re-excision for positive margins, those who underwent re-excision for positive or close margins after adjuvant therapy, and those with missing or incomplete information. Overall, 542 patients with pT1–T3 breast cancer were included in this analysis. According to the final margin status after definitive therapy, the patients were divided into two groups: the closed (group A) and negative (group B) groups. Patients who did not undergo re-excision for close (≤2 mm) margins were classified into group A. In contrast, patients with negative (>2 mm) margins after the initial BCS and those who underwent re-excision for positive or close margins to obtain negative margins were classified into group B (Figure 1).

Preoperative tumor localization was performed for non-palpable lesions. Ultrasonography (US)-guided hook wire localization was used for US-detectable lesions, and mammography (MMG)-guided hook wire localization was used for microcalcifications that could be visualized with only MMG. We did not use charcoal for tumor localization. A standardized intraoperative specimen orientation marking system was routinely performed using sutures and clips, following the hospital protocol. Specimen MMG was performed to confirm complete removal of microcalcifications in the case of lesions with only microcalcifications or tumors with suspected extratumoral microcalcifications. Intraoperative frozen sections were obtained for margin assessment in patients with suspected inadequate margins. A multidisciplinary tumor board comprising breast surgeons, pathologists, medical oncologists, radiologists, and radiation oncologists made all the treatment decisions.

Tumors were histologically classified according to the World Health Organization guidelines. Surgical margin status was assessed by determining the microscopic distance between tumor cells and the edge of the specimen in millimeters. The final surgical margin width was equal to the closest margin in any direction for either invasive carcinoma or ductal carcinoma in situ (DCIS). Cylindrical removal of the breast tissue from the skin to the pectoral fascia was performed in most cases. Re-excision was not recommended for positive or close superficial and deep margins. Therefore, the superficial and deep margin statuses were not considered in this analysis. A positive margin referred to the presence of in situ or invasive cancer cells at the margin of the resected tissue. In contrast, a close margin status referred to the presence of cancer cells ≤2 mm from the resected margin but not at the margin itself. 

LRR was defined as an invasive or non-invasive relapse in the ipsilateral breast and axillary lymph nodes. The following variables were considered to be associated with LRR: age, histological type of positive or close margin (DCIS or invasive component), tumor size, lymph nodal status, histology, grade according to Elston and Ellis, multifocality, extensive intraductal component (EIC), lymphovascular invasion (LVI), hormone receptor status, human epidermal growth factor receptor 2 (HER2) status, and Ki-67 expression. Subgroup analysis was performed to identify the factors related to residual disease in patients who underwent re-excision. 

Data were analyzed using SPSS software (version 25.0; IBM, Armonk, NY, USA). Baseline comparisons between the different groups are reported with medians corresponding to ranges or absolute and relative frequencies, depending on the underlying scale level of the variable. Group comparisons were performed using the chi-square test for categorical variables and the Mann–Whitney U test for continuous variables. The 10-year rate of patients experiencing LRR was estimated using the Kaplan–Meier estimation model, and the log-rank test was used to compare the two groups. The factors influencing LRR were identified using univariable and multivariable Cox models. The 95% confidence intervals (CIs) for the corresponding hazard ratios (HRs) are reported. Statistical significance was set at *p* < 0.05. 

This study was approved by the institutional review board of KHUMC (IRB No. 2021-12-028). 

## 3. Results

During the study period, 751 women with breast cancer underwent BCS, of whom 542 were patients with pT1–T3 who met the inclusion criteria of our study. The participants’ baseline characteristics are presented in Table 1. The median patient age was 53 (interquartile range [IQR], 46–62) years. A total of 384 (70.8%) patients had (y)pT1 disease, and 424 (78.2%) had (y)pN0 disease. A total of 371 (68.5%) patients had negative resection margins, and 171 (31.5%) had positive or close resection margins after the initial BCS. Multifocality and EIC were observed in 73 (13.5%) and 116 (21.4 %) patients, respectively. Moreover, 435 (80.3%) patients had hormone receptor-positive breast cancer, and 414 (76.4%) had HER2-negative breast cancer. Most patients received adjuvant RTx and systemic therapy according to the tumor stage, hormone receptor status, and HER2 status.

A total of 428 (79.0%) patients had negative resection margins and 114 (21.0%) had close resection margins after definitive surgery. The clinicopathological characteristics according to the final margin status after definitive surgery are listed in Table 2. Group B was younger than group A (*p* = 0.011). Approximately 47% of group A had a high Ki-67 proliferative index, whereas 30.1% of group B had a high Ki-67 index. There were no differences in tumor size, nodal status, histology, grade, multifocality, EIC, LVI, hormone receptor status, HER2 status, adjuvant RTx, and systemic therapy between the two groups.

**Figure 2:**
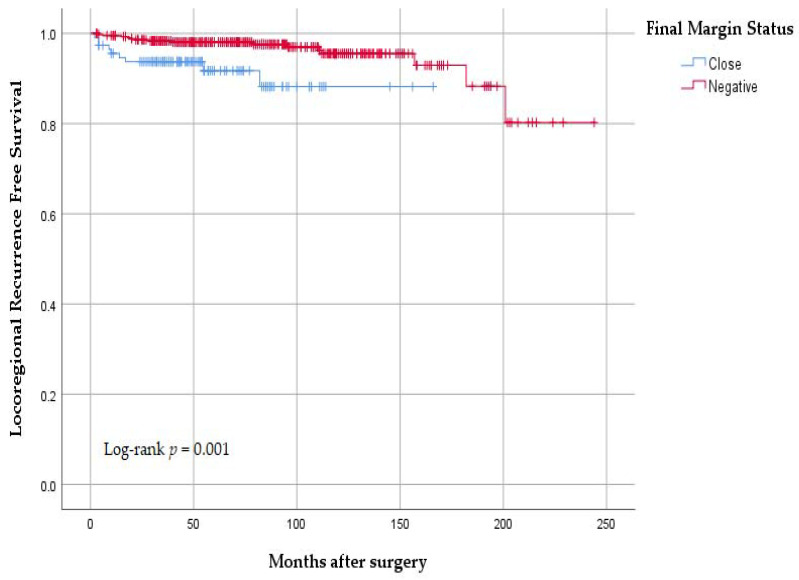
Locoregional recurrence-free survival according to final margin status after definitive surgery.

The median follow-up period was 72 (IQR, 42–113) months. A total of 24 (4.4%) patients developed LRR: 9 in group A and 15 in group B. The 10-year LRR-free survival rates were 88.2% and 95.5%, respectively (*p* = 0.001) (Figure 2).

Table 3 presents the results of multivariable Cox proportional hazards regression analyses for LRR. RTx was associated with a reduced LRR risk (HR, 0.127; 95% CI, 0.038–0.421). Patients with negative margins after definitive surgery had a lower risk of LRR than those with close margins (HR, 0.215; 95% CI, 0.086–0.543).

Of the 57 patients who underwent re-excision for either positive or close margins, 34 (59.6%) had evidence of residual cancer in the re-excision specimen, whereas the remaining 23 (40.4%) had no residual disease. Residual disease was found in 24 (70.6%) patients with positive margins and in 10 (29.4%) patients with close margins. Multivariable analysis showed that multifocality was an independent predictor of residual cancer (odds ratio [OR], 10.580; 95% CI, 1.840–60.843). In addition, patients with invasive components at positive or close margins were less likely to have residual disease upon re-excision than those with DCIS components at positive or close margins (OR, 0.140; 95% CI, 0.022–0.904) (Table 4).

## 4. Discussion

Inadequate margin status after BCS is associated with increased LR and distant recurrence [19]. Since most local failures develop at the previous resection site, they can be attributed to residual disease after initial BCS [15]. Recent international guidelines suggest that adequate margins for invasive cancer should be *no ink on tumor* after BCS [10]. However, the residual cancer risk after re-excision of a close margin is reportedly equal to that after resection of a positive margin [20]. Re-excision should be considered in cases of positive and close margins with a high probability of residual disease around the surgical cavity. Therefore, predicting the presence of residual disease is important to identify patients with positive or close margins who have a high risk of local failure after BCS. In a meta-analysis of margin status after BCS, the pooled estimate for the prevalence of a *tumor on ink* margin was 9.4% (95% CI 6.8%–12.8%), and that of a tumor within 2 mm of the inked margin was 17.8% (95% CI 13.0%–23.9%) [19]. In this study, 38 (7.0%) patients had positive resection margins after the initial surgery. Our low positive margin rate may have resulted from improved preoperative imaging, which determines the disease extent accurately. To assess the extent of the lesions requiring resection, we routinely performed magnetic resonance imaging (MRI), MMG, and US preoperatively (MRI was performed in 90.2% of patients; MMG, in 97.6%; and US, in 99.8%). MRI was not performed for only 9.8% of patients for the following reasons: absolute contraindications to MRI and non-cooperation. These imaging techniques aided in the selection of BCS-suitable patients. In addition, when necessary, we performed intraoperative margin assessments, such as frozen section and specimen MMG. Therefore, only 57 patients underwent re-excision for positive or close margins. 

Previous studies have reported 21–77% as the rate of residual disease after re-excision, with young age, large tumor size, EIC, multifocality, DCIS component at positive or close margins, and node positivity as factors associated with it [20,21,22,23,24,25,26,27]. In this study, residual cancer after re-excision was found in 34 (59.6%) patients, and multifocality was significantly correlated with the presence of residual disease. Consistent with our results, Sabel et al. demonstrated that multifocality was an independent predictive factor of residual disease, and that the presence of residual disease in re-excisions for close and positive margins were equal [20]. Gurdal et al. also reported that approximately 77% of patients with multifocality had residual disease, and that most patients with multifocality had residual disease regardless of their margin status (positive margin, 86.4% vs. negative close margin, 85.7%) [28]. These results suggest that re-excision of close margins should be strongly considered for multifocal breast cancer. 

Although multifocality has been identified as a predictor of residual disease after BCS, some authors have reported that aggressive surgery for multifocality did not improve outcomes in terms of locoregional or distant recurrence. Neri et al. reported that multifocal cancers were related to higher locoregional and distal relapse rates than unifocal cancers, regardless of the type of surgery performed, and that the median breast cancer-specific survival for multifocality did not differ between patients treated with BCS (177.4 months; 95% CI, 150.3–204.5) and those treated with mastectomy (145.2 months; 95% CI, 128.6–161.7; *p*  =  0.148) [29]. In our study, multifocality did not affect LRR. This may be due to the fact that there were only 17 patients with multifocality who did not undergo re-excision for close margins.

According to the National Comprehensive Cancer Network guidelines, even for invasive cancers with a component of DCIS, *no ink on tumor* is recommended for either DCIS or invasive cancer cells as an adequate margin, as the natural history and outcomes of these lesions are more similar to invasive cancer than to DCIS [30]. Interestingly, our results showed that positive or close margins bordering the DCIS component were associated with residual disease after the initial BCS. Generally, DCIS may have a multifocal distribution with a gap. Therefore, even a negative margin does not guarantee the absence of cancer cells in the remnant breast tissue [31]. Although recent guidelines suggest that patients with invasive cancer with a DCIS component should be treated on the basis of invasive cancer recommendations, individual assessments may be used in clinical decision-making for re-excision. 

The goal of adjuvant RTx is to eradicate any tumor deposits that may persist after BCS or mastectomy. Adjuvant RTx reduces the risk of LRR and breast cancer mortality [32]. A previous study demonstrated that the LR rate after BCS followed by whole-breast radiation therapy (WBRT) was only approximately 5% after a median follow-up period of 79.2 months [10]. Therefore, WBRT is recommended for most patients who undergo BCS. In this study, we found that patients receiving adjuvant RTx had an almost eight-fold lower risk of LRR than patients without adjuvant RTx. 

An involved or close margin is one of the most important risk factors for LR. Compared with a negative margin, it is associated with an approximately two-fold increase in risk [10,33]. The high local failure rate was attributed to high rates of residual disease associated with inadequate resection margins. In several studies, re-excision of positive and close margins was associated with a decreased risk of local failure [34,35,36,37]. Importantly, this study found that a close margin was a prognostic factor for LRR. Similar to our results, those of a recent meta-analysis suggested that surgeons should aim to obtain a minimum clear margin of at least 1 mm, with the authors recommending the reappraisal of international guidelines [19].

Changes in the understanding of breast cancer biology and the widespread use of adjuvant systemic therapy for early breast cancer have also affected attitudes toward margins [38]. The National Surgical Adjuvant Breast and Bowel Project B-24 trial showed that the adjuvant tamoxifen reduced ipsilateral breast tumor recurrence rates in patients with positive margins to levels similar to those in patients with negative margins [39]. Thus, routine re-excision of close margins is not mandatory in the era of multidisciplinary treatment. Nevertheless, our results indicate that close margins after BCS are an important prognostic factor for LRR, suggesting that individual assessment of recurrence and additional appropriate treatment are required in these patients.

Our analysis was limited by the low recurrence rates and its retrospective design. Because of the low event rate, the power to detect statistically significant differences between the two groups in the survival analysis was insufficient. In addition, because we did not perform additional surgery in patients with negative (>2 mm) margins, we could not evaluate the rate of unrecognized residual disease. Further studies with larger cohorts are needed to assess the impact of margin status after BCS on oncologic outcomes.

## 5. Conclusions

A close resection margin after BCS is associated with an increased LRR risk in patients with invasive breast cancer. We recommend additional treatment for patients with close margins and a high risk of residual disease to ensure optimal oncologic outcomes after an informed discussion between surgeons and patients with full disclosure of the increased risk of recurrence associated with close margins.

## Figures and Tables

**Table 1:** Patients’ clinicopathological characteristics.

Variables	Values, n (%)
Age, years	
≤40	52 (9.6%)
41–60	343 (63.3%)
≥61	147 (27.1%)
Margin status after initial BCS	
Positive	38 (7.0%)
Close, ≤2 mm	133 (24.5%)
Negative	371 (68.5%)
Final margin status after definitive surgery	
Close, ≤2 mm	114 (21.0%)
Negative	428 (79.0%)
Tumor size	
≤2 cm	384 (70.8%)
>2 cm	158 (29.2%)
Nodal status	
Negative	424 (78.2%)
Positive	118 (21.8%)
Histology	
IDC	456 (84.1%)
ILC	15 (2.8%)
Mixed/Other	71 (13.1%)
Grade	
I	150 (27.7%)
II	201 (37.1%)
III	154 (28.4%)
Unknown	37 (6.8%)
Multifocality	
Absent	469 (86.5%)
Present	73 (13.5%)
EIC	
Absent	426 (78.6%)
Present	116 (21.4%)
LVI	
Absent	437 (80.6%)
Present	105 (19.4%)
Hormone receptor	
Negative	107 (19.7%)
Positive	435 (80.3%)
HER2	
Negative	414 (76.4%)
Positive	96 (17.7%)
Unknown	32 (5.9%)
Ki-67	
<15	197 (36.3%)
≥15	182 (33.6%)
Unknown	163 (30.1%)
Neoadjuvant CTx	
No	518 (95.6%)
Yes	24 (4.4%)
Adjuvant CTx	
No	264 (48.7%)
Yes	278 (51.3%)
Adjuvant HTx	
No	113 (20.8%)
Yes	429 (79.2%)
Adjuvant HER2-targeted therapy	
No	477 (88.0%)
Yes	65 (12.0%)
Adjuvant RTx	
No	23 (4.2%)
Yes	519 (95.8%)
Types of recurrence	
Locoregional	24 (4.4%)
Distant	29 (5.4%)
Any	42 (7.7%)

BCS, breast-conserving surgery; IDC, invasive ductal carcinoma; ILC, invasive lobular carcinoma; EIC, extensive intraductal component; LVI, lymphovascular invasion; HER2, human epidermal growth factor receptor 2; CTx, chemotherapy; HTx, hormone therapy; RTx, radiation therapy.

**Table 2:** Patients’ clinicopathological characteristics according to final margin status after definitive surgery.

Variables	Group A (%) n = 114	Group B (%) n = 428	*p*-Value ^1^
Age, years			0.011
≤40	4 (3.5%)	48 (11.2%)	
41–60	70 (61.4%)	273 (63.8%)	
≥61	40 (35.1%)	107 (25.0%)	
Tumor size			0.056
≤2 cm	89 (78.1%)	295 (68.9%)	
>2 cm	25 (21.9%)	133 (31.1%)	
Nodal status			0.472
Negative	92 (80.7%)	332 (77.6%)	
Positive	22 (19.3%)	96 (22.4%)	
Histology			0.787
IDC	94 (82.5%)	362 (84.6%)	
ILC	4 (3.5%)	11 (2.6%)	
Mixed/Other	16 (14.0%)	55 (12.8%)	
Grade			0.627
I	35 (30.7%)	115 (26.9%)	
II	42 (36.8%)	159 (37.1%)	
III	32 (28.1%)	122 (28.5%)	
Unknown	5 (4.4%)	32 (7.5%)	
Multifocality			0.611
Absent	97 (85.1%)	372 (86.9%)	
Present	17 (14.9%)	56 (13.1%)	
EIC			0.877
Absent	89 (78.1%)	337 (78.7%)	
Present	25 (21.9%)	91 (21.3%)	
LVI			0.807
Absent	91 (79.8%)	346 (80.8%)	
Present	23 (20.2%)	82 (19.2%)	
Hormone receptor			0.146
Negative	28 (24.6%)	79 (18.5%)	
Positive	86 (75.4%)	349 (81.5%)	
HER2			0.180
Negative	93 (81.6%)	321 (75.0%)	
Positive	18 (15.8%)	78 (18.2%)	
Unknown	3 (2.6%)	29 (6.8%)	
Ki-67			<0.001
<15	46 (40.4%)	151 (35.3%)	
≥15	53 (46.5%)	129 (30.1%)	
Unknown	15 (13.2%)	148 (34.6%)	
Neoadjuvant CTx			1.000 ^2^
No	109 (95.6%)	409 (95.6%)	
Yes	5 (4.4%)	19 (4.4%)	
Adjuvant CTx			0.602
No	58 (50.9%)	206 (48.1%)	
Yes	56 (49.1%)	222 (51.9%)	
Adjuvant HTx			0.061
No	31 (27.2%)	82 (19.2%)	
Yes	83 (72.8%)	346 (80.8%)	
Adjuvant HER2-targeted therapy			0.234
No	104 (91.2%)	373 (87.1%)	
Yes	10 (8.8%)	55 (12.9%)	
Adjuvant RTx			0.600
No	6 (5.3%)	17 (4.0%)	
Yes	108 (94.7%)	411 (96.0%)	

SD, standard deviation; BCS, breast-conserving surgery; DCIS, ductal carcinoma in situ; IDC, invasive ductal carcinoma; ILC, invasive lobular carcinoma; EIC, extensive intraductal component; LVI, lymphovascular invasion; HER2, human epidermal growth factor receptor 2; CTx, chemotherapy; HTx, Hormone therapy; RTx, radiation therapy. ^1^ Chi-square test; ^2^ Fisher’s exact test.

**Table 3:** Cox proportional hazard model of risk factors associated with locoregional recurrence.

Variables	Univariable		Multivariable	
	HR	95% CI	HR	95% CI
Age, years				
≤40	1			
41–60	0.454	0.141–1.461		
≥61	1.330	0.391–4.522		
Tumor size				
≤2 cm	1		1	
>2 cm	0.888	0.350–2.252	0.752	0.288–1.966
Nodal status				
Negative	1		1	
Positive	1.258	0.498–3.177	1.911	0.715–5.106
Histology				
IDC/ILC	1			
Mixed/Other	1.870	0.696–5.025		
Grade				
I	1			
II	0.713	0.250–2.034		
III	0.985	0.345–2.810		
Multifocality				
Absent	1		1	
Present	0.273	0.037–2.023	0.298	0.039–2.286
EIC				
Absent	1			
Present	1.225	0.484–3.096		
LVI				
Absent	1			
Present	1.329	0.490–3.606		
Hormone receptor				
Negative	1		1	
Positive	0.505	0.207–1.232	0.535	0.203–1.412
HER2				
Negative	1		1	
Positive	1.506	0.541–4.191	1.645	0.572–4.728
Ki-67				
<15	1			
≥15	1.367	0.417–4.481		
Adjuvant RTx				
No	1		1	
Yes	0.147	0.054–0.401	0.127	0.038–0.421
Final margin status after definitive surgery				
Close	1		1	
Negative	0.262	0.109–0.633	0.215	0.086–0.543

HR, hazard ratio; CI, confidence interval; BCS, breast-conserving surgery; DCIS, ductal carcinoma in situ; IDC, invasive ductal carcinoma; ILC, invasive lobular carcinoma; EIC, extensive intraductal component; LVI, lymphovascular invasion; HER2, human epidermal growth factor receptor 2; RTx, radiation therapy.

**Table 4:** Logistic regression analysis of predictive factors associated with residual disease in patients who underwent re-excision.

Variables	Univariable		Multivariable	
	OR	95% CI	OR	95% CI
Age, years				
≤40	1			
41–60	1.857	0.330–10.446		
≥61	0.833	0.114–6.111		
Margin status after initial BCS				
Positive	1			
Close	0.648	0.212–1.979		
Histological type of positive or close margin				
DCIS component	1		1	
Invasive component	0.508	0.173–1.490	0.140	0.022–0.904
Tumor size				
≤2 cm	1			
>2 cm	1.181	0.360–3.871		
Nodal status				
Negative	1			
Positive	1.979	0.536–7.309		
Histology				
IDC/ILC	1			
Mixed/Other	0.788	0.506–1.227		
Grade				
I	1			
II	1.083	0.279–4.210		
III	1.650	0.370–7.365		
Multifocality				
Absent	1		1	
Present	4.667	1.159–18.783	10.580	1.840–60.843
EIC				
Absent	1		1	
Present	0.330	0.108–1.009	0.232	0.038–1.406
LVI				
Absent	1			
Present	2.591	0.715–9.387		
Hormone receptor				
Negative	1			
Positive	2.083	0.494–8.782		
HER2				
Negative	1			
Positive	1.333	0.223–7.980		
Ki-67				
<15	1		1	
≥15	4.407	1.074–18.092	4.815	0.976–23.756
Neoadjuvant CTx				
No	1			
Yes	0.202	0.020–2.077		

OR, odds ratio; CI, confidence interval; BCS, breast-conserving surgery; DCIS, ductal carcinoma in situ; IDC, invasive ductal carcinoma; ILC, invasive lobular carcinoma; EIC, extensive intraductal component; LVI, lymphovascular invasion; HER2, human epidermal growth factor receptor 2; CTx, chemotherapy.

## Data Availability

The data presented in this study are available on request from the corresponding author.
